# Universal Health Coverage for Antiretroviral Treatment: A Review

**DOI:** 10.3390/idr15010001

**Published:** 2022-12-21

**Authors:** Aklilu Endalamaw, Charles F Gilks, Fentie Ambaw, Tesfa Dejenie Habtewold, Yibeltal Assefa

**Affiliations:** 1 School of Public Health, The University of Queensland, Brisbane, QLD 4072, Australia; 2 College of Medicine and Health Sciences, Bahir Dar University, Bahir Dar P.O. Box 79, Ethiopia; 3 School of Public Health, College of Medicine and Health Sciences, Bahir Dar University, Bahir Dar P.O. Box 79, Ethiopia; 4 Branch of Epidemiology, Division of Population Health Research, Division of Intramural Research, Eunice Kennedy Shriver National Institute of Child Health and Human Development, National Institutes of Health, Bethesda, MD 20892, USA; 5 Department of Epidemiology, University Medical Center Groningen, University of Groningen, 9712 CP Groningen, The Netherlands

**Keywords:** coverage, equity, financial protection, quality of care, universal health coverage

## Abstract

Universal health coverage is essential for the progress to end threats of the acquired immunodeficiency syndrome epidemic. The current review assesses the publication rate, strategies and barriers for antiretroviral therapy (ART) coverage, equity, quality of care, and financial protection. We searched Web of Science, PubMed, and Google Scholar. Of the available articles, 43.13% were on ART coverage, 40.28% were on financial protection, 10.43% were on quality of care, and 6.16% were on equity. A lack of ART, fear of unwanted disclosure, lack of transportation, unaffordable health care costs, long waiting time to receive care, and poverty were barriers to ART coverage. Catastrophic health care costs were higher among individuals who were living in rural settings, walked greater distances to reach health care institutions, had a lower socioeconomic status, and were immunocompromised. There were challenges to the provision of quality of care, including health care providers’ inadequate salary, high workload and inadequate health workforce, inappropriate infrastructure, lack of training opportunities, unclear division of responsibility, and the presence of strict auditing. In conclusion, ART coverage was below the global average, and key populations were disproportionally less covered with ART in most countries. Huge catastrophic health expenditures were observed. UHC contexts of ART will be improved by reaching people with poor socioeconomic status, delivering appropriate services, establishing a proper health workforce and service stewardship.

## 1. Introduction

Antiretroviral therapy (ART) is effective [1] in controlling viral replication and preventing acquired immunodeficiency syndrome (AIDS), which is a global threat of epidemic diseases. AIDS killed an estimated 650,000 people globally in 2021 [2]. It is the second leading cause of disability-adjusted life years among people aged 25 to 49 years [3]. 

Ending the global threat of AIDS epidemic is a global development goal. The United Nations (UN) member states set seventeen goals in which universal health coverage (UHC) and ending AIDS epidemic targets are integrated [4]. UHC is an action framework that builds on effective service coverage and financial protection [5]. Effective service coverage refers to (1) reproductive, maternal, and child health, (2) infectious diseases, (3) non-communicable diseases, and (4) service provision and access [6]. Financial protection comprises catastrophic health expenditure (CHE), impoverishment, and poverty due to health care costs [7]. Quality of care and equity of services are integrated in the UHC definition [8].

Equitable service, provision of quality care [9], and affordable health care [2] could reflect the accelerated progress of UHC. People living with HIV (PLHIV) should start ART based on a standardized treatment protocol [10]. It is about “providing evidence-based health care services to those who need them, avoiding harm to people for whom the care is intended, and providing care that responds to individual preferences, needs and values” [11]. Especially, providing high-quality HIV/AIDS services in low- and middle-income countries is critical [12]. Quality service delivery should not vary by age, gender, race/ethnicity, geographical location, religion, socioeconomic status, or linguistic and political affiliation [13]. There have been strategies to minimize service disparity, including mobilizing resources, establishing an ethics advisory body, creating opportunities for public dialogue, developing and implementing policies, and identifying vulnerable and marginalized populations [14]. 

UNAIDS has recommended conducting research on different HIV/AIDS issues [15]. Identifying gaps in the current research knowledge guides future research area [16]. Available scoping reviews [17,18] did not assess the publication rate on ART coverage, equity, quality of care, and financial protection. The current review is important to synthesize these concepts based on the available literature. Available fragmented evidence around the world is inconclusive and disaggregated. Therefore, the current review reasonably assessed ART coverage, financial protection, equity, and the quality of care; together, these dimensions are intimately linked and integrated in the UHC concept. The current review will provide evidence for policy makers and be used as a framework for researchers to conduct further research because this review is the first of its kind to incorporate four major domains into a single health topic. 

## 2. Materials and Methods 

### 2.1. Theoretical Framework

This review was conducted based on the UHC concept: “Universal health coverage means that all people have access to the health services they need, when and where they need them, without financial hardship” [19]. From this notion, dimensions of UHC are described as:

- Coverage: who is covered? This concerned crude coverage in the current review;
- Financial protection: the proportion of the costs covered, and service costs covered by insurance or other methods. The effectiveness of financial protection is measured by catastrophic health expenditure and impoverishment;
- Equity in ART coverage: all people in need of service should be covered; the non-covered population is evidence of service disparity;
- Quality in ART care: in UHC, effective service coverage denotes the quality of services delivered to the people covered by services. Quality of care was assessed by any of methods in this review. Quality of care and equity are integrated definitions of UHC.

### 2.2. Identifying a Research Question

The study was conducted using a population, concept, and context framework in a scoping review methodology [20,21,22]. In the current review, HIV/AIDS patients (population), coverage, equity, financial protection and quality of care (concept) and studies were not limited to a specific country (context). 

The topic is registered in Open Science Framework (OSF|Universal Health Coverage for Antiretroviral Treatment: a scoping review). The steps started with the identification of a review question followed by setting inclusion criteria, selecting articles, extracting data, synthesizing, and reporting. The review was reported in accordance with the Preferred Reporting Items for Systematic reviews and Meta-analysis (PRISMA) extension for scoping reviews checklist [23]. 

### 2.3. Searching, Selection, Charting and Presenting

Web of Science, PubMed, and Google Scholar were searched between 20 October 2021 and 12 November 2021, and updated on 3 March 2022. A UNAIDS 2021 report on ART coverage was used to see the difference between researchers’ findings and the panel data report [24]. Search terms were “universal”, “health”, “health care”, “healthcare”, “health service”, “quality”, “access”, “coverage”, “equity”, “disparity”, “inequity”, “equality”, “inequality”, “expenditure”, “cost”, “HIV”, “human immunodeficiency virus”, AIDS, “acquired immunodeficiency syndrome”, “HIV/AIDS”, and “human immunodeficiency virus/acquired immunodeficiency syndrome”. “AND” or “OR” Boolean operators were used to broaden and narrow search results. Search strategy of each database is shown in supplementary file (Table S1). Articles were imported in EndNote desktop version x7 for duplication removal and citation. An automatic duplication check was performed using EndNote desktop version x7 proceeding with manual duplication removal. 

Studies published in English language were included. To access research post-2015 sustainable development target 3.8, the search was restricted to include studies conducted after 2015. Non-English language, abstract only, comments to editor, erratum, perspectives, letters, and brief communications were excluded. While counting articles based on key terms, barriers, and enablers in a publication rate for each dimension, further screening was conducted to describe ART coverage (inequity) and/or strategies and barriers, catastrophic health expenditure and/or determinants (challenges) and quality of care and/or strategies and barriers. Costs or cost-effectiveness of ART, out-of-pocket expenditure, health expenditure, insurance coverage, and catastrophic health expenditure were considered in the publication description of financial protection. Quality of care was extracted when the article’s objective was on the quality of ART care or quality of care among HIV/AIDS clients in general (some did not specify ART). Inequity was disparity between countries, ethnic groups, citizen, and non-citizen and/or socioeconomic or demographic differences in ART use. 

Titles, abstracts, and full texts of articles were reviewed in a stepwise approach. A piloted and refined data extraction tool was used to extract data. Data were scrutinized, charted, and tabulated based on key themes and findings. The information extracted was on author(s), publication year, World Health Organisation (WHO) regions, World Bank (WB) group, study methods, and main findings. 

Available articles were compiled and summarized with frequency and percentage based on the information extracted. The types of study design were quantitative, qualitative, and mixed research. Simple descriptive analysis was carried out, and the results are presented in figures, tables, and text. 

## 3. Results 

### 3.1. Search Results

**Figure 1:**
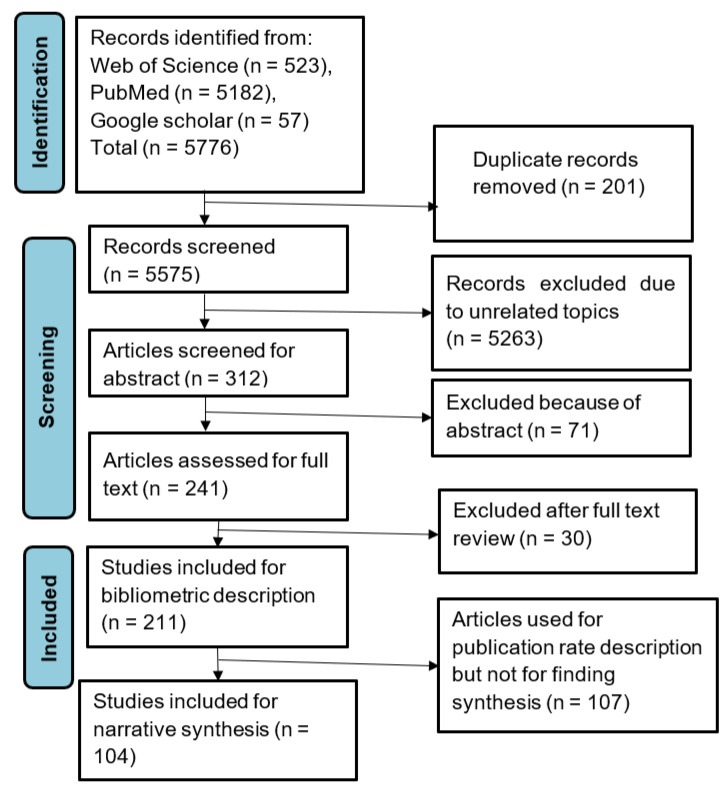
PRISMA flow diagram for the article selection process.

**Figure 2:**
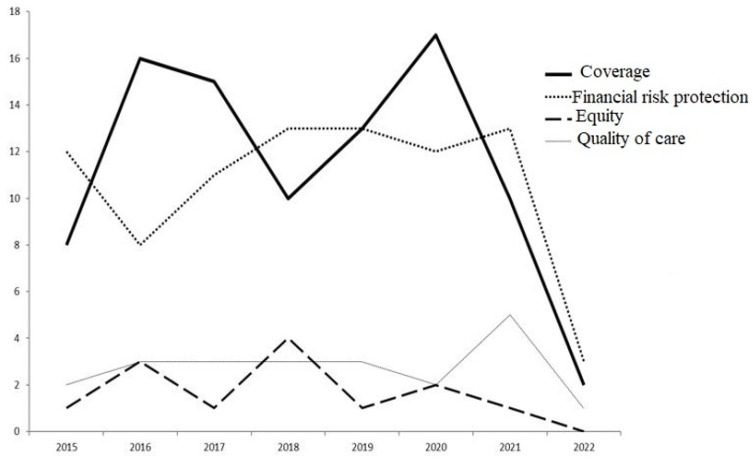
Research production of UHC dimensions on ART from January 2015 to March 2022.

The selection of the studies is depicted in Figure 1. The publishing rates for ART coverage, equity, financial protection, and quality of care were examined across 211 articles. To describe ART coverage or equity, catastrophic health expenditure and quality of care, as well as strategies and challenges or determinants for each, 104 of these were included. In the Supplementary Materials (Table S2), all 211 articles’ attributes are displayed. The majority (91 articles) focused on ART coverage, followed by 85 articles on reducing financial risk protection; 22 articles were on quality of care, and 13 articles were on equity. Figure 2 shows the publishing rate for each topic. 

### 3.2. Main Findings

The following text and figures summarize the main findings on ART coverage, equity, quality of care, and catastrophic health expenditure; the UNAIDS reports for ART coverage corresponding to available articles for all ages or key populations are described.

### 3.3. ART Coverage among PLHIV

**Figure 3:**
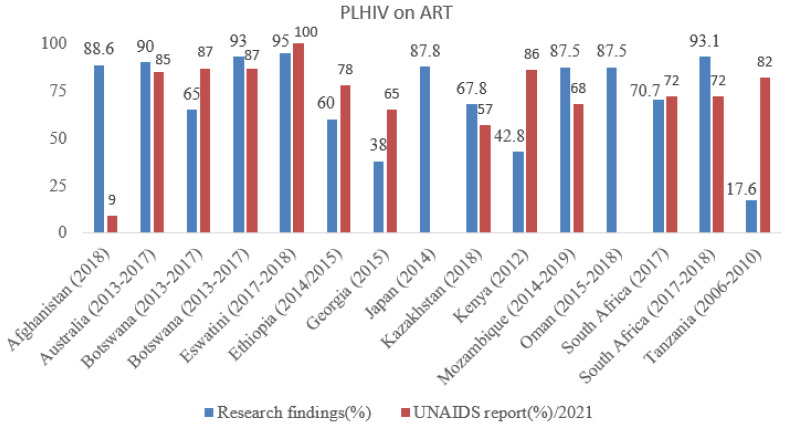
ART coverage based on research findings by survey year and UNAIDS 2021 report for all age groups.

The number of PLHIV who were on ART is reported; in Oman, 1682 PLHIV were on ART, whereas in China, 978,138 were; however, there were no percentage data for Oman or Japan in the 2021 UNAIDS report. The findings of the research and the UNAIDS report varied for most countries. For instance, Afghanistan (88.6% in research versus 9% in UNAIDS report) and Kazakhstan (67.8% versus 57%) exhibited disparity between subnational and national estimates. ART coverage differed between two studies conducted in the same country (65% versus 93% in Botswana and 70.7% versus 93.1% in South Africa), showing discrepancy between different settings and/or time periods (Figure 3).

### 3.4. ART Coverage among Key Population

**Figure 4:**
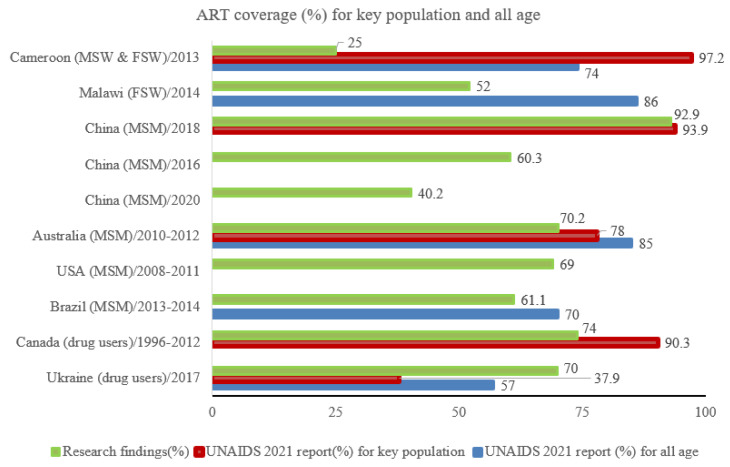
Percentage of ART coverage among key populations (drug users, female sex workers/FSW, and male who has sex with male/MSM) based on available research with survey year and the 2021 UNAIDS report for key population and all ages.

Of eight countries which had research findings on key populations, UNAIDS did not report on Brazil, the USA, or Malawi. In Ukraine, ART coverage among injection drug users was higher based on research finding (70%) than the UNAIDS report (37.9%); ART coverage was lower in this group than the general population (37.9% versus 57%). In Canada, 74% of injection drug users were on ART in 2012 (based on research) and 90.3% in 2021 (based on UNAIDS report), which highlights changing coverage over time (Figure 4).

### 3.5. Catastrophic Health Expenditure

**Figure 5:**
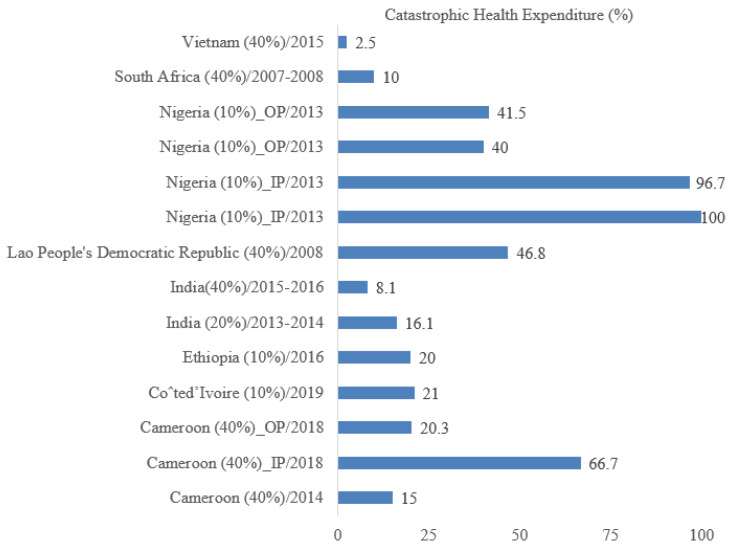
Percentage of catastrophic health expenditure in different countries based on threshold (10% and 40%), the department (IP: inpatient and OP: outpatient), and survey year.

Catastrophic health spending was significantly higher for inpatient care than outpatient services. It was 66.7% for inpatients and 20.3% for outpatients in Cameroon, and it was 100% (inpatient) versus 40% (outpatient) in Nigeria (Figure 5).

### 3.6. Strategies for ART Coverage and Quality of Care

Community-based HIV prevention and treatment interventions (including home-based care and family strategy) were effective in expanding ART coverage and improving the quality of care. Strengthening financial and leadership activities increased ART coverage. Strengthening health workforce (e.g., training and increasing numbers) improved the quality of care (Table 1).

### 3.7. Barriers for ART Coverage, Catastrophic Health Expenditure, and Quality of Care

The lack of ART was a barrier to ART coverage in some countries. Other obstacles to extending ART and raising the standard of service were the workload faced by health care providers, stigma, and discrimination. Transportation problems were a barrier for ART coverage and catastrophic health expenditure (Table 2).

### 3.8. Client (Sociodemographic- and Clinical-Related) Factors

Women, urban residents, and non-immigrants had higher ART coverage rates compared with their counterparts. Those who were living in rural residences, had a lower socioeconomic status, were far from health service areas, and were immunosuppressed (lower CD4 cell counts) were at risk of catastrophic healthcare costs. Those living in rural areas and who had attended ART for more than ten years responded that the service they were receiving was of poor quality (Table 3).

## 4. Discussion

In this analysis, it was discovered that the high HIV risk group (MSM and FSW) had a lower ART coverage. There was a discrepancy between the national and subnational estimates of ART coverage. Catastrophic health spending was higher. A community-based HIV prevention and treatment program was successful in increasing access to ART and raising the standard of care. Leadership initiatives and financial stabilization boosted ART coverage. Strengthening the health staff increased the standard of care. Financial strengthening and leadership activities increased ART coverage. Health workforce strengthening improved the quality of care. The lack of ART was a barrier to ART coverage in some nations. Another obstacle in expanding ART and raising the standard of service was the workload placed on medical professionals. Difficulties with transportation were a barrier to ART coverage and catastrophic medical costs. Compared with their counterparts, women, urban residents, and non-immigrants had higher ART coverage rates. Those with lower socioeconomic positions, a rural residence, a greater distance from a health care region, and with a reduced immune system were subject to catastrophic health costs. Rural residents who had been receiving ART for more than ten years stated that the care they were receiving was of poor quality. The equity dimension was the least investigated topic.

The current review found that 90% or more of PLHIV who were aware of their HIV status had started ART in South Africa [66], Eswatini [67], Botswana [68], and Australia [69], all of which reported achieving their ART coverage goals. This does not imply that all of these nations are successful in providing ART to individuals in need. In South African research, for instance, only a small number of patients were tested for HIV infection, allowing ART to be provided to those few instances and resulting in a high rate of ART coverage. Peer-to-peer community-based HIV testing and the index-client testing approach were put into practice in Eswatini. Similarly, the “Botswana Combination Prevention Project” was an interventional trial conducted in Botswana. This project’s goal was to identify HIV-positive individuals who were not receiving ART and refer them to the ART clinics. Due to these circumstances, PLHIV were forced to start receiving ART in Botswana and Eswatini. Universal ART coverage in Australia reached 95%, but migrant populations had a lower ART coverage. Nearly all children with HIV were receiving ART in France, which is also making excellent progress [70]. This was explained by the fact that France has successfully implemented new HIV/AIDS policies [71]. In Oman [72], Afghanistan [73], Japan [74], and Mozambique [75], ART coverage was likewise reported to be higher than the global average point. The high rate of late HIV diagnosis in Oman may be the source of the reasonable rate of ART coverage, because to the accompanying opportunistic diseases, commencing ART is more likely if PLHIV were recently identified. Additionally, since 2015, the test and treat strategy may improve ART coverage in Oman, Afghanistan, and Mozambique.

A test and treat strategy on MSM was effective in China [76]. The 2021 UNAIDS report, however, showed a different result from the original finding in some countries. This could be because, first, many UNAIDS reports were HIV treatment cascades (percentage of PLHIV who started ART among all PLHIV), whereas most original research reported above 95% (the proportion of PLHIV who had started ART among PLHIV who knew their HIV status in the survey year). Second, the estimates in the UNAIDS study are modelled at the national level, which can occasionally differ from the actual issue at the regional or local level. Third, there is a current coverage gap between a particular research area and the national estimate (e.g., in Kazakhstan and South Africa). Fourth, the 2021 UNAIDS reports cover every age group, whereas some studies exclusively include adults (e.g., a study in Kazakhstan). Finally, there was variation in the survey year; ART coverage was higher in 2020 than it was in 2010 [24]. However, Georgia [77], Tanzania [78], and another study of South Africa [79] exhibited lower ART coverage rates. Before using the test and treat method, studies were carried out in Tanzania and Georgia. In South Africa, this was conducted among adolescent girls and young women. This age group has low ART coverage in most nations. Likewise, ART coverage was low among key populations (MSM, FSW, and drug users). These population groups experience significant levels of stigma or prejudice, which resulting in the reduced utilization of health service [80,81]. In both UNAIDS and research, key population’s ART coverage status was not well reported. There are countries where huge numbers of FSW with high HIV rates are living, but their ART coverage status is not recognized. For example, one out of five FSW had a confirmed diagnosis for HIV infection in Ethiopia [82], indicating a need for critical and urgent policy intervention.

Intervention programs in the community and at home help PLHIV to begin ART. When health services are embedded in the community, transportation costs are reduced, enabling patients to more readily afford HIV/AIDS-related services [83]. Membership to clubs and peer support groups raised awareness levels, offered emotional support, and helped with other administrative issues [84]. One of the key takeaways from the 2022 International AIDS Society conference conducted in Barcelona was that “communities with PLHIV continue to lead the way” because they can alter the community viewpoint in policy formulation and implementation [85].

A substantial proportion of PLHIV suffer from CHE, with the burden differing between nations. Financial assistance and insurance increased access to ART, but a greater percentage of PLHIV was prone to high health care expenses in Côte d’Ivoire [86], Ethiopia [87], Nigeria [58], India [62], and the Lao People’s Democratic Republic [64]. These numbers were unacceptably high for PLHIV who lived in rural areas, were far from a healthcare facility, had lower monthly income, and were illiterate and unemployed. The standard of HIV/AIDS care was also impaired by microeconomic factors [88]. Financial assistance for the poor, which includes increasing the country’s health insurance coverage, is an important strategy [38].

CHE and poverty were less well researched topics in financial protection. Less research was conducted on the equity dimension. In a similar vein, a bibliometric examination of HIV research found that equity concerns have received the least attention [89]. This might be due to researchers, the health department, and both public and commercial organizations being interested in determining the percentage of PLHIV on ART. This might motivate researchers to conduct more studies on ART coverage than equity. Despite this and the global push to achieve ART coverage, the required level of ART coverage has not yet been met, except in a few countries. This situation might encourage organizations to pay more attention to how social factors affect the equality of HIV/AIDS services [90].

## 5. Conclusions

Most studies concentrate on ART coverage and financial protection, while less evidence was on equity and quality of care. ART coverage was lower among the high HIV risk group, and disparities in ART coverage were common based on equity dimensions (ethnicity, sex, residence, and immigration status). People living with HIV in different countries were exposed to catastrophic costs, which were significantly higher among the poor, rural populations, and those living far from HIV clinics. Community-based interventions (peer and community support, home-based/door-to-door support, and membership) were effective strategies to improving ART coverage and the quality of care; effective leadership (presence of laws and monitoring) and financial strengthening (e.g., insurance) improved ART coverage. Health workforce strengthening improved the quality of care. Policy- and decisionmakers should consider challenges related to socioeconomic status, service delivery, health workforce, financing, and leadership if countries are really wanting to realize UHC.

## Figures and Tables

**Table 1:** Strategies (✓) that enhance the ART coverage and quality of care.

Strategies	Coverage	Quality of Care
Diagnosed after the country’s change in treatment approach [25]	✓	
Community-based HIV prevention and treatment interventions (test and treat) [26,27,28,29,30]	✓	
Peer (group) and community support [31,32,33]	✓	✓
Home-based care, including door-to-door communication [32,34,35]	✓	
Family health strategy [36]		✓
Being a member of adherence clubs of HIV-positive people [37]	✓	
Health insurance [38]	✓	
Food security [38]	✓	
Government expenditure on health [39]	✓	
Effective governance [39]	✓	
Virology testing by 2 months of age [39]	✓	
Density of healthcare workers per 10,000 population [39]	✓	
Presence of laws to combat barriers [31]	✓	
Ensuring regular internal mentorship [40,41]	✓	✓
Availing supplies [40]		✓
Providing refresher training [40]		✓
Physician network connectedness [42]		✓
Comfort in the clinic [43]		✓

**Table 2:** Barriers (✓) for ART coverage, catastrophic health expenditure and quality of care.

Barriers	Coverage	Catastrophic Health Expenditure	Quality of Care
Logistical problems [44]	✓		
ART shortage [31,44,45]	✓		
Workload [41,44,46]	✓		✓
Shortage of trained health force [41,46]			✓
Poverty [47]	✓		
Stigma and discrimination [31,43,46]	✓		✓
Long waiting times in ART clinic [31]	✓		
Transportation unavailability [31]	✓		
Cost problems [31]	✓		
Clients fear of unwanted disclosure [45]	✓		
Mode of transportation (motor cycle) than private car [48]		✓	
Implementation of the free ART policy in middle income [49]		✓	
Lack of confidentiality [43]			✓
Legal prosecution [43]			✓
Poor infrastructure [41]			✓
Unclear division of responsibility between health care providers [46]			✓
Limited training opportunity [46]			✓
Strict auditing [46]			✓

**Table 3:** Socio-demographic and clinical-related factors (✓) for ART coverage, catastrophic health expenditure, and quality of care.

Determinants	More Covered (Inequity)	High Catastrophic Health Expenditure	Poor Quality of Care
Caregivers aged 40–49 years [38]	✓		
Older [50,51]	✓		✓
Age 15–19 years [52]	✓		
Age 18–25 and 36–45 years [51]	✓		✓
Female [51,52,53]	✓		✓
White vs. Black [50,54,55,56]	✓		
Urban dwellers [38,57]	✓		
Rural residence [58,59,60,61]		✓	✓
Lives far distance from health care settings [62,63,64]		✓	
Divorced adult [48]		✓	
Non-immigrants vs. immigrants [50]	✓		
Educated [38]	✓		
Being unemployed [63]		✓	
Lower socioeconomic class [59,62,63,64]		✓	
Belonged to nuclear family [63]		✓	
Non-disabled vs. disabled [38]	✓		
Live in high HIV burden area [65]	✓		
Acquired HIV through sexual transmission rather than injection drug users [25]	✓		
Diagnosed in later clinical stages [25]	✓		
Had a CD4 cell count lower than 200 [63]		✓	
Attending clinic more than 10 years [51]			✓
Visiting clinics every 3 months [51]			✓

## Data Availability

All the required data are available within the manuscript and supplementary files.

## Supplementary Materials

The following supporting information can be downloaded at: https://www.mdpi.com/article/10.3390/idr15010001/s1, Table S1: Search strategy, Table S2: Characteristics of articles.
