# Supplementary material for: Universal Health Coverage for Antiretroviral Treatment: A Review
Source: Infect Dis Rep. 2022 Dec 21;15(1):1–15. doi: 10.3390/idr15010001 (PMC9844463; doi:10.3390/idr15010001)
Supplement: Supplementary file 1 [file idr-15-00001-s001.zip › idr-2023105-supplementary.pdf]

Table S1. Search strategy.

Database	Table S1-Search strategy	Result	Filters applied:
PubMed	(coverage[Title/Abstract] OR equity[Title/Abstract] OR disparity[Title/Abstract] OR inequity[Title/Abstract] OR equality[Title/Abstract] OR inequality[Title/Abstract] OR expenditure [Title/Abstract] OR cost [Title/Abstract) AND (("hiv treatment" [Title/Abstract] OR "human immunodeficiency virus treatment"[Title/Abstract] OR "AIDS treatment" [Title/Abstract] OR "acquired immunodeficiency syndrome treatment"[Title/Abstract] OR "hiv/aids treatment" [Title/Abstract] OR "human immunodeficiency virus /acquired immunodeficiency syndrome treatment"[Title/Abstract] OR ART[Title/Abstract] OR "antiretroviral therapy"[Title/Abstract] OR “antiretroviral treatment"[Title/Abstract])) NOT (Address OR Autobiography OR Bibliography OR Clinical conference OR Clinical Trail Protocol OR Clinical Trial, Veterinary OR Comment OR Editorial OR Duplicate Publication OR Guideline OR Interactive Tutorial OR Retracted Publication OR Published Erratum OR Letter OR Lecture	5182	From 2015/1/1 - 2022/3/3

	OR News) Filters applied: from 2015/1/1 - 2022/3/3		
Web of Science	(((TI=(coverage OR equity OR disparity OR inequity OR equality OR inequality OR quality OR expenditure OR cost))) AND ((TI=(“HIV treatment” OR "human immunodeficiency virus treatment " OR “AIDS treatment” OR "acquired immunodeficiency syndrome treatment " OR “HIV/aids treatment” OR "human immunodeficiency virus /acquired immunodeficiency syndrome treatment " OR ART OR “antiretroviral therapy” OR “antiretroviral treatment”))) AND (PY==("2021" OR "2020" OR "2019" OR "2018" OR "2016" OR "2015" OR "2017"))) NOT Document Types: Letters or Meeting Abstracts or Editorial Materials or Corrections or Book chapters or Books or Book reviews or Biographical-Items or News Items or Proceedings Papers or Retractions or Data Papers	523	From 2015/1/1 - 2022/3/3

Table S2: Characteristics of articles

Author	Publication year	Country	WHO region	World Bank economy	Study approach	Specific term	Major category
Bachanas P et al	2021	Botswana	Africa	LM	Quantitative	coverage	Coverage

Kebaabetsw P et al	2020	Botswana	Africa	Upper middle	Quantitative	Barriers to access	Coverage
Mathews C et al	2021	South Africa	Africa	Upper middle	Quantitative	ART coverage	Coverage
Mnyaka OR et al	2021	South Africa	Africa	Upper middle	Quantitative	Barriers to treat	Coverage
Plazy M et al	2016	South Africa	Africa	Upper middle	Quantitative	ART coverage	Coverage
Puttkammer N et al	2020	Haiti	Americas	LM	Quantitative	ART coverage	Coverage
Levira F et al	2015	Tanzania	Africa	LM	Quantitative	coverage	Coverage
Castillo-Cañón JC et al	2021	Colombia	Americas	Upper middle	Quantitative	Healthcare-related expenditures	Financial protection
Etiaba E et al	2016	Nigeria	Africa	LM	Quantitative	catastrophic health expenditure	Financial protection
Barennes H et al	2015	Lao People's Democratic Republic	Western Pacific	LM	Quantitative	OOP & CHE	Financial protection
Aliyu A et al	2019	Nigeria	Africa	LM	Quantitative	Quality	Quality
Alvi Y et al	2020	India	South-East Asia	LM	Quantitative	OOP & CHE	Financial protection
Assefa Y et al	2020	Across countries	Across countries	Across	Mixed method (Literature & USAID/WHO data)	ART coverage & its inequity	inequity
Cunnama L et al	2018	Swaziland	Africa	LM	Quantitative	Cost effectiveness	Financial protection
de Freitas MA et al	2016	Brazil	Americas	Upper middle	Quantitative	ART coverage	Coverage
Dickerson S et al	2020	Malawi	Africa	low	Quantitative	medical spending, capacity to pay and catastrophic health expenditure	Financial protection

Exavery A et al	2020	Tanzania	Africa	LM	Quantitative	expenditures	Coverage
Genberg BL et al	2019	Kenya	Africa	LM	Quantitative	ART coverage	Coverage
McCreesh N et al	2017	Uganda	Africa	low	Quantitative	Cost effectiveness	Financial protection
Miyano S et al	2017	Zambia	Africa	LM	Quantitative	Cost effectiveness	Financial protection
Nosyk B et al	2015	Canada	Americas	High	Quantitative	cost effectiveness	Financial protection
Taverne B et al	2021	Senegal	Africa	LM	Quantitative	OOP & CHE	Financial protection
Teeraananchai et al	2020	Thailand	South-East Asia	Upper middle	Quantitative	ART coverage	Coverage
Vu L et al	2016	Uganda	Africa	low	Quantitative	cost	Financial protection
Vu LTH et al	2021	Vietnam	Western Pacific	LM	Quantitative	OOP & CHE	Financial protection
Were LPO et al	2020	Kenya	Africa	LM	Quantitative	Insurance	Financial protection
McClarty LM et al	2021	Canada	Americas	High	Quantitative	sociodemographic	Inequity
Yakob BC	2015	Ethiopia	Africa	low	Quantitative	Quality	Quality
Visser CA	2018	South Africa	Africa	Upper middle	Mixed method	Quality	Quality
Stecher C et al	2021	USA	Americas	High	Quantitative	Quality	Quality
Seeberger CA	2019	Uganda	Africa	low	Quantitative	Quality	Quality
Rice WS et al	2020	USA	Americas	High	Qualitative	Quality	Quality
Rewari BB et al	2017	India	South-East Asia	LM	Quantitative	Quality	Quality

Rawat A et al	2018	South Africa	Africa	Upper middle	Quantitative	Quality	Quality
Rabkin M et al	2017	Kenya	Africa	LM	Quantitative	Quality	Quality
Pilgrim N et al	2018	Zambia	Africa	LM	Qualitative	Quality	Quality
Orlando S et al	2016	Malawi	Africa	low	Quantitative	Cost effectiveness	Financial protection
Olivieri-Mui B et al	2021	USA	Americas	High	Quantitative	Quality	Quality
Nduaguba SO et al	2020	USA	Americas	High	Quantitative	Cost	Financial protection
MacKenzie LJ et al	2017	Canada	Americas	High	Quantitative	Quality	Quality
Luna-Mireles et al	2019	Mexico	Americas	Upper middle	Quantitative	Quality	Quality
Li C et al	2020	China	Western Pacific	Upper middle	Qualitative	Quality	Quality
Landovitz RJ et al	2016	USA	Americas	High	Quantitative	Quality	Quality
Kesselring S et al	2016	Canada	Americas	High	Quantitative	Quality	Quality
Karver TS et al	2022	Dominance Republic	Across countries	Across	Mixed	Quality	Quality
Ghate M et al	2015	India	South-East Asia	LM	Quantitative	Quality	Quality
do Nascimento L et al	2016	Brazil	Americas	Upper middle	Quantitative	Quality	Quality
Cholong BJ et al	2022	Cameroon	Africa	LM	Quantitative	CHE	Financial protection
Chaumont C et al	2019	Dominance Republic	Across countries	Across	Quantitative	OOP expenditure	Financial protection
Bousmah M et al	2021	Cameroon	Africa	LM	Quantitative	CHE	Financial protection
Assebe LF et al	2020	Ethiopia	Africa	low	Quantitative	CHE	Financial protection

Nelson RE et al	2018	USA	Americas	High	Quantitative	Healthcare expenditures	Financial protection
Negin J et al	2017	South Africa	Africa	Upper middle	Quantitative	CHE	Financial protection
Ndukwe CD et al	2018	Nigeria	Africa	LM	Quantitative	OOP expenditure	Financial protection
Long LC et al	2016	South Africa	Africa	Upper middle	Quantitative	Cost of ART	Financial protection
Lee E et al	2021	Australia	Western Pacific	High	Quantitative	OOP expenditure	Financial protection
Forsythe SS et al	2019	Across countries	Across countries	Across	Quantitative	Cost of ART	Financial protection
Stelmach RD et al	2021	Coˆted'Ivoire	Africa	LM	Quantitative	OOP expenditure	Financial protection
Shukla M et al	2015	India	South-East Asia	LM	Quantitative	CHE	Financial protection
Setiawan E et al	2022	Indonesia	South-East Asia	LM	Quantitative	Coverage & Cost for ART	Coverage
Onwujekwe OE et al	2016	Nigeria	Africa	LM	Quantitative	Healthcare expenditures	Financial protection
Tran BX et al	2016	Vietnam	Western Pacific	LM	Quantitative	Socioeconomic disparity	Inequity
Moyo S et al	2018	Kenya & South Africa	Africa	Across	Quantitative	Socio-demographic	Inequity
Laut KG et al	2018	Across countries	European	Across	Quantitative	three HIV key populations	Inequity
Girum T et al	2018	Ethiopia	Africa	low	Quantitative	Gender	Inequity
Auld AF et al	2015	Across countries	Across countries	Across	Quantitative	Gender	Inequity

Adeyinka DA et al	2017	Sub-Saharan Africa	Africa	Across	Quantitative	ART Coverage	Coverage
Chan BT et al	2015	Sub-Saharan Africa	Africa	Across	Quantitative	ART coverage	Coverage
Castro R et al	2016	Brazil	Americas	Upper middle	Quantitative	ART coverage	Coverage
Burrage A et al	2018	Sub-Saharan Africa	Africa	Across	Quantitative	ART coverage	Coverage
Beer L et al	2016	USA	Americas	High	Quantitative	Race, ethnicity, gender	Inequity
Barnabas RV et al	2016	South Africa & Uganda	Africa	Across	Quantitative	ART coverage	Coverage
Barlow-Mosha L et al	2017	Across countries	Across countries	Across	Review	Risks & benefit of Universal ART	Coverage
Aung NHHL et al	2020	Myanmar	South-East Asia	LM	Mixed	ART coverage	Coverage
Astawesegn FH et al	2022	Sub-Saharan Africa	Africa	Across	Quantitative	ART coverage	Coverage
Assefa Y et al	2019	Ethiopia	Africa	low	Mixed (review & quantitative, qualitative)	ART coverage	Coverage
Assefa Y et al	2017	Ethiopia	Africa	low	Mixed (review & quantitative, qualitative)	ART coverage	Coverage
Goga AE et al	2017	Across countries	Across countries	Across	Review	ART coverage	Coverage
Gebre Y et al	2016	Caribbean	Across countries	Across	Review	ART coverage	Coverage
García-Deltoro M	2019	USA	Americas	High	Review	ART coverage	Coverage
Furuoka F et al	2015	Sub-Saharan Africa	Africa	Across	Quantitative	Determinants	Coverage
Floyed S et al	2020	Zambia & South Africa	Africa	Across	Quantitative	ART coverage	Coverage
Estill J et al	2018	Sub-Saharan Africa	Africa	Across	Quantitative	ART coverage	Coverage
Elgalib A et al	2020	Oman	Eastern Mediterranean	High	Quantitative	ART coverage	Coverage
Dutta A et al	2015	Across countries	Across countries	Across	Quantitative	Cost to scale-up ART	Financial

							protection
Demeke HB et al	2020	USA	Americas	High	Quantitative	disparity ethnic	Inequity
Dahourou DL	2017	Sub-Saharan Africa	Africa	across	Review	challenges & compliance ART coverage	Coverage
King EJ et al	2021	Kazakhstan	European	Upper middle	Quantitative	ART coverage	Coverage
Kim AA et al	2016	Kenya	Africa	LM	Quantitative	ART coverage	Coverage
Indravudh PP et al	2021	Malawi	Africa	LM	Quantitative	ART coverage	Coverage
Huerga H et al	2018	South Africa	Africa	Upper middle	Quantitative	ART coverage	Coverage
Hoots BE et al	2017	USA	Americas	High	Quantitative	ART coverage	Coverage
Holland CE et al	2015	Cameroon	Africa	LM	Quantitative	ART coverage	Coverage
Hayes R et al	2017	Zambia	Africa	LM	Quantitative	ART coverage	Coverage
Harooni MZ et al	2021	Afghanistan	Eastern Mediterranean	low	Quantitative	ART coverage	Coverage
Haacker M	2018	Kenya	Africa	LM	Quantitative	Barriers to access	Coverage
Lou J et al	2017	China	Western Pacific	Upper middle	Quantitative	ART coverage	Coverage
Lolekha R et al	2020	Thailand	South-East Asia	Upper middle	Quantitative	ART coverage	Coverage
Levi J et al	2016	Across countries	Across countries	Across	Review	ART coverage	Coverage

Lebelonyane R et al	2021	Botswana	Africa	Upper middle	Quantitative	ART coverage	Coverage
Lazuardi E et al	2020	Indonesia	South-East Asia	LM	Qualitative	ART coverage	Coverage
Larmarange J et al	2018	South Africa	Africa	Upper middle	Quantitative	ART coverage	Coverage
Lancaster KE et al	2016	Malawi	Africa	low	Quantitative	ART coverage	Coverage
De La Mata NL et al	2015	Australia	Western Pacific	High	Quantitative	ART coverage	Coverage
Pathmanathan I et al	2020	Mozambique	Africa	low	Quantitative	ART coverage	Coverage
Oklakunde BO et al	2019	Sub-Saharan Africa	Africa	Across	Quantitative	ART coverage	Coverage
New TW et al	2021	Myanmar	South-East Asia	LM	Quantitative	ART coverage	Coverage
Miyazaki N et al	2017	Japan	Western Pacific	High	Quantitative	ART coverage	Coverage
Mendez-Lopez A et al	2019	Across countries	Across countries	Across	Quantitative	ART coverage	Coverage
Mboup A et al	2018	Benin	Africa	LM	Quantitative	ART coverage	Coverage
Marukutira T et al	2020	Australia	Western Pacific	High	Quantitative	ART Coverage	Coverage
Marinda E et al 2020	2020	South Africa	Africa	Upper middle	Quantitative	ART coverage	Coverage
Maman D et al	2016	Malawi	Africa	low	Quantitative	ART coverage	Coverage
Makhema J et al	2019	Botswana	Africa	Upper middle	Quantitative	ART coverage	Coverage
Tao L et al	2017	China	Western Pacific	Upper middle	Quantitative	ART Coverage	Coverage
Takarinda KC et al	2016	Zimbabwe	Africa	LM	Quantitative	ART Coverage	Coverage
Soumah A et al	2019	France	European	High	Quantitative	ART Coverage	Coverage
Solomon SS et al	2016	India	South-East Asia	LM	Quantitative	ART Coverage	Coverage

Shen M et al	2018	USA	Americas	High	Quantitative	cost effectiveness	Financial protection
Shanaube K et al	2020	Zambia & South Africa	Africa	Across	Quantitative	ART Coverage	Coverage
Scott N et al	2017	Australia	Western Pacific	High	Quantitative	ART Coverage	Coverage
Sazonova Y et al	2020	Ukraine	European	LM	Quantitative	ART Coverage	Coverage
Saito S et al	2018	Across countries	Africa	Across	Quantitative	ART Coverage	Coverage
Sabapathy K et al	2017	Zambia & South Africa	Africa	Across	Quantitative	ART Coverage	Coverage
Zelaya CE et al 2016	2016	Vietnam	South-East Asia	LM	Quantitative	RCT-ART Coverage	Coverage
Yoong D et al	2018	Canada	Americas	High	Quantitative	OOP expenditure	Financial protection
Yang X et al	2020	China	Western Pacific	Upper middle	Quantitative	ART Coverage	Coverage
Yan I et al	2016	41 high HIV-TB burden countries	Across countries	Across	Quantitative	ART Coverage	Coverage
Wirth KE et al	2020	Botswana	Africa	Upper middle	Quantitative	ART Coverage	Coverage
Williams D et al	2021	Eswatini	Africa	LM	Quantitative	ART Coverage	Coverage
Vu LTH et al	2020	Vietnam	Western Pacific	LM	Quantitative	OOP expenditure	Financial protection
Zhuang X et al	2018	China	Western Pacific	Upper middle	Quantitative	ART Coverage	Coverage
Beck E et al	2021	Croatia	European	High	Quantitative	cost of ART care	Financial protection
Ross EL et al	2015	USA	Americas	High	Quantitative	cost effectiveness of ART care	Financial protection
Siregar AYM et al	2015	Indonesia	South-East Asia	LM	Quantitative	cost of treatment	Financial protection
Belay YB et al	2021	Ethiopia	Africa	low	Quantitative	cost-utility	Financial protection

Naidoo K et al	2015	South Africa	Africa	Upper middle	Quantitative	cost-effectiveness	Financial protection
Duffy M et al	2019	Uganda, South Africa, Zimbabwe	Africa	Across	Qualitative	Enablers and Barriers	Coverage
Maddali MV et al	2015	India	South-East Asia	LM	Quantitative	Cost effectiveness	Financial protection
Culhane J et al	2020	Kenya	Africa	LM	Quantitative	cost effectiveness	Financial protection
Ouattara EN et al	2019	Côte d'Ivoire	Africa	LM	Quantitative	cost effectiveness	Financial protection
Krentz H et al	2019	Canada	Americas	High	Quantitative	cost effectiveness	Financial protection
Nichols BE et al	2021	Zambia	Africa	LM	Quantitative	costs	Financial protection
Wu H et al	2021	China	Western Pacific	Upper middle	Quantitative	cost	Financial protection
Gumede-Moyo S et al	2019	Zambia	Africa	LM	Quantitative	ART Coverage	Coverage
Johnson LF et al	2020	South Africa	Africa	Upper middle	Quantitative	ART Coverage	Coverage
Desmonde S et al	2019	South Africa, cote d'ivoire & Burkina Faso	Africa	across	Quantitative	cost effectiveness of ART care	Financial protection
Mikkelsen E et al	2017	Ghana	Africa	LM	Quantitative	cost of ART care	Financial protection
Estrada V et al	2022	Spain	European	High	Quantitative	cost	Financial protection
Thin K et al	2019	Cambodia	South-East Asia	LM	Quantitative	cost	Financial protection
Tucker A et al	2020	Zambia	Africa	LM	Quantitative	cost	Financial protection

Ong KJ et al	2019	British	European	High	Quantitative	cost	Financial protection
Dugdale CM et al	2019	South Africa	Africa	Upper middle	Quantitative	cost effectiveness	Financial protection
Treskova M et al	2016	Germany	European	High	Quantitative	cost	Financial protection
Zheng A et al	2018	India	South-East Asia	LM	Quantitative	cost	Financial protection
Boerma R et al	2019	Sub-Saharan Africa	Africa	Across	Quantitative	ART Coverage	Coverage
Sharma A et al	2019	India	South-East Asia	LM	Quantitative	cost	Financial protection
Chkartishvili N et al	2016	Georgia	European	Upper middle	Quantitative	ART Coverage	Coverage
Safarnejad A	2017	Across countries	Across countries	Across	Review	Enablers and Barriers	Coverage
Thuppal SV et al	2015	India	South-East Asia	LM	Quantitative	cost	Financial protection
Wu C et al	2021	China	Western Pacific	Upper middle	Quantitative	ART Coverage	Coverage
Agarwal R et al	2017	India	South-East Asia	LM	Quantitative	cost	Financial protection
Uthman R et al	2018	Sub-Saharan Africa	Africa	across	Quantitative	cost	Financial protection
Hu Q et al	2019	China	Western Pacific	Upper middle	Quantitative	cost	Financial protection
Mbachu C et al	2016	Nigeria	Africa	LM	Qualitative	Political economy of ART decentralization	Coverage
Kumar GA et al	2018	India	South-East Asia	LM	Quantitative	cost	Financial protection

Odayar J et al	2019	South Africa	Africa	Upper middle	Quantitative	ART coverage	Coverage
Johns B et al	2017	Vietnam	Western Pacific	LM	Quantitative	OOP	Financial protection
Larson BA et al	2018	Kenya	Africa	LM	Quantitative	cost	Financial protection
Papot E et al	2017	France	European	High	Quantitative	cost	Financial protection
Joseph B et al	2016	Canada	Americas	High	Quantitative	ART coverage	Coverage
Ciaranello AL et al	2015	Africa	Africa	Across	Quantitative	Cost effectiveness	Financial protection
Cornelius LJ et al	2018	Nigeria	Africa	LM	Qualitative	Barriers to access	Coverage
Oddershede L et al	2016	British	European	High	Quantitative	Cost effectiveness	Financial protection
Jamieson L et al	2021	South Africa	Africa	Upper middle	Quantitative	cost & cost-effectiveness	Financial protection
Tweya H et al	2016	Malawi	Africa	low	Quantitative	cost effectiveness	Financial protection
Bhatasara S	2015	Zimbabwe	Africa	low	Quantitative	ART coverage	Coverage
Jain V et al	2015	Uganda	Africa	low	Quantitative	Costs	Financial protection
Enns B et al	2019	USA	Americas	High	Quantitative	costs ART on vs ART off	Financial protection
Ritchwood TD et al	2017	USA	Americas	High	Quantitative	Expenditure	Financial protection
Punekar YS et al	2019	China	Western Pacific	Upper middle	Quantitative	ART coverage	Coverage
Beer L et al	2016	USA	Americas	High	Quantitative	Race, gender, Ethnicity	Inequity

Maggiolo F et al	2015	Italy	European	High	Quantitative	cost	Financial protection
Amzel A et al	2018	South Africa	Africa	Upper middle	Quantitative	ART coverage	Coverage
Boyer S et al	2020	Burkina Faso, Cameroon, Senegal	Africa	Across	Quantitative	cost effectiveness	Financial protection
Church K et al	2017	Sub-Saharan Africa	Africa	across	Quantitative	Factors	Coverage
Restelli U et al	2017	Italy	European	High	Quantitative	Cost effectiveness	Financial protection
Parker B et al	2021	Canada	Americas	High	Quantitative	cost effectiveness	Financial protection
Taramasso L et al	2018	Italy	European	High	Quantitative	cost	Financial protection
Shrestha RK et al	2020	USA	Americas	High	Quantitative	Cost	Financial protection
McCann NC et al	2020	USA	Americas	High	Quantitative	cost	Financial protection
Kimaro GD et al	2017	Tanzania	Africa	LM	Quantitative	cost	Financial protection
Adjetery V et al	2019	Ghana	Africa	LM	Qualitative	access	Coverage
Barry M et al	2022	Saudi Arabia	Eastern Mediterranean	High	Quantitative	cost	Financial protection
Tierrablanca LE et al	2018	11 countries	Across countries	Across	Quantitative	cost effectiveness	Financial protection
Garay OU et al	2019	France	European	High	Quantitative	Cost effectiveness	Financial protection
Alhassan RK et al	2021	Ghana	Africa	LM	Quantitative	Quality of ART care	Quality
Yeshiwas Y et al	2021	Ethiopia	Africa	low	Quantitative	Quality of ART care	Quality

Julien A et al	2021	South Africa	Africa	Upper middle	Qualitative	Quality of ART care	Quality
Steinert JI et al	2021	Eswatini	Africa	LM	Quantitative	CHE	Financial protection
Zhang L et al	2015	Thailand	South-East Asia	Upper middle	Quantitative	ART coverage	Coverage
Granich R et al	2015	30 countries	Across countries	Across	Quantitative	ART coverage	Coverage
Long LC et al	2017	South Africa	Africa	Upper middle	Quantitative	Cost effectiveness	Financial protection
Kalichman SC et al	2017	USA	Americas	High	Quantitative	ART coverage	Coverage
Landovitz RJ et al	2017	USA	Americas	High	Quantitative	Race	Inequity
Llibre JM et al	2018	Spain & France	European	High	Quantitative	Cost effectiveness	Financial protection
Grimes RM et al	2018	USA	Americas	High	Quantitative	cost	Financial protection
Laut K et al	2018	European countries	European	Across	Quantitative	Disparity b/n countries	Inequity
Mao JB et al	2019	USA	Americas	High	Quantitative	Cost	Financial protection
Valbert F et al	2020	Germany	European	High	Quantitative	Cost	Financial protection
Marukutira T et al	2019	Botswana	Africa	Upper middle	Quantitative	citizen vs non-citizen	Inequity

CHE: Catastrophic Health Expenditure; LM; lower-middle; OOP: Out-of-pocket
